# The Disposal and Treatment of the Insane

**Published:** 1895-02-09

**Authors:** Edwin Goodall

**Affiliations:** Assistant Medical Officer, West Riding Asylum, Wakefield


### THE DISPOSAL AND TREATMENT OF THE INSANE.

By Edwin Goodall, M.D.Lond., B.S., M.R.C.P., Assistant Medical Officer, West Riding Asylum, Wakefield.

At the Newcastle meeting of the British Medical Association, Dr. Hack Tuke, in closing his observations upon the question of the increase of insanity, made the dispiriting admission that, notwithstanding all our efforts, the cure of insanity remains as distant as ever. This statement can scarcely be contraverted. Experience of county asylums, whilst showing that the recovery-rate (which cannot, of course, be designated the *cure*-rate) remains practically stationary, brings out the disturbing fact that the duration of life in the incurable cases is greater. Various reasons, which may be summed up under the term "improved hygiene," co-operate to produce the latter result. From the point of view of common humanity it may be thought undesirable that this should be spoken of as "disturbing"; by extending one's conception of humanity, however, so as to include the rights of the majority, one can absolve oneself from any charge of inhumanity in contemplating without self-reproach the more speedy demise of these unfortunates. It is, indeed, a question of the best disposal of attention and space; and in this case—as in that of wounded from the battle-field—the needs of the curable are paramount.

The candid alienist would probably admit that he derives a sort of dog-in-the-manger satisfaction from the reflection that the cure of certain common somatic diseases appears as remote as that of certain forms of insanity. It is doubtless true that, in the whole range of mental disorders there is none amenable to positive, undisputed cure, in the sense that scabies or chlorosis is. But can it be said that the cure of acute delirious mania or melancholia is more distant than that of acute lobar pneumonia or acute nephritis? The pathogenesis is as obscure and the treatment as diverse in the one case as in the other. The claim of superior knowledge (and hence greater likelihood of cure in the latter instance) cannot even be raised upon the basis of a pathogenic organism, for if pneumonia has its diplo-coccus, acute delirious mania has its bacillus (see "Rasori. Centralbl.," f. Bakt. xiv., B. No. 16), and the one is as likely to prove the *causa vera* as the other.

Only too frequently the alienist is discouraged at the very outset of treatment by the knowledge that the case is one of hereditary insanity. To take an instance from recent experience: a patient is admitted in a state of melancholia; after careful examination, a good prognosis seems justified. On inquiring into the history, however, it transpires that the patient's mother was a melancholiac, and died in the asylum, weak-minded; the maternal grandfather committed suicide by drowning, a maternal aunt is at present insane, and two maternal uncles are habitual drunkards. Often the history is worse, there being insanity on the father's side also. Temporary recovery, speedy relapse, chronic insanity (rendering the patient a burden to the community for years)—this is the gloomy vista opened up by the contemplation of such a case. The stamp of degeneracy is upon these unfortunates, and to attempt to cure them is to fly in the face of nature. It is with relief we turn to others, in which, at any rate, there is a negative history of heredity; in some of these that factor may be excluded with as much confidence as it is possible to entertain in regard to any disease. This class may be described as the pick of the asylum, and the practical question arises, should they be segregated and receive greater individual attention? This is one of the most important questions now agitating the asylum world. The writer is cordially with those who advocate the separate treatment of the recent and curable cases. At present they are thrust hurriedly and indiscriminately into the asylum ark, and too often thrust out of sight—lost in the dreary waste of incurability. The asylum physician, be he never so conscientious, cannot be expected to attend adequately to the hopeful cases, merged as they are in a seething mass of dementia and imbecility. Mere segregation of the acute cases is not all that is desired. The acute hospital should have an adequate medical staff, hampered as little as possible by clerical work, and not at all by supervision of dements. The provision for the latter should be of the simplest kind; they can dispense with the well-appointed quarters and the lavish medical attendance they at present receive. Blue Books and medical literature will be none the poorer if the clerical labour at present bestowed on such cases be cut down wholesale. Foolscap was never put to more absurd use, nor energy more wantonly wasted. Let the energy and the skill be freely spent upon the cases with potentiality of sound recovery and useful work in the world. The hum of progress may then replace the silence of stagnation, even in asylums.

Far greater than that just considered is the question of the prevention of insanity. We can scarcely, perhaps, be blamed for leaving its solution to generations unborn. It is even more a legal than a medical question. Whether the restrictions and penalties, the enforcement of which a radical consideration of this matter would entail, would ever be tolerated in a free country such as ours, is extremely doubtful. It is likely that the first attempts to solve this problem will be made by one of the Continental States.

